# Retrospective Clinicopathological Analysis of 65 Peri-Implant Lesions

**DOI:** 10.3390/medicina57101069

**Published:** 2021-10-07

**Authors:** Amir Shuster, Gal Frenkel, Shlomi Kleinman, Oren Peleg, Clariel Ianculovici, Eitan Mijiritsky, Ilana Kaplan

**Affiliations:** 1 Department of Otolaryngology, Head and Neck Surgery and Maxillofacial Surgery, Tel-Aviv Sourasky Medical Center, Tel Aviv 64239, Israel; gfrenk@gmail.com (G.F.); shlomik@tlvmc.gov.il (S.K.); orenp@tlvmc.gov.il (O.P.); clarielc@tlvmc.gov.il (C.I.); mijiritsky@bezeqint.net (E.M.); 2 Department of Oral and Maxillofacial Surgery, Goldschleger School of Dental Medicine, Tel-Aviv University, Tel Aviv 39040, Israel; 3 Department of Oral Pathology, Oral Medicine and Maxillofacial Radiology Goldschleger School of Dental Medicine, Tel-Aviv University, Tel Aviv 39040, Israel; dr.ilanakaplan@gmail.com; 4 Pathology Department, Sackler School of Medicine, Tel-Aviv University, Tel Aviv 39040, Israel

**Keywords:** peri-implantitis, histology, pathology, giant cell granuloma, pyogenic granuloma

## Abstract

*Background and Objectives*: Peri-implantitis is a common finding among patients with dental implants. There is no consensus regarding the treatment of this disease, but in many cases, surgical treatment is common practice. A histopathological analysis is not an integral part of suggested protocols. The present study investigated the clinical and histopathological parameters of lesions mimicking peri-implantitis and correlated them with the outcome and follow-up data. *Materials and Methods*: The study included 65 consecutive biopsies taken from peri-implantitis patients between 2008–2019. *Results*: The three common diagnoses were fibro-epithelial hyperplasia 20 (30.7%), pyogenic granuloma 16 (24.6%), and peripheral giant cell granuloma 15 (23%). There were 18 cases of recurrent lesions in the study group (27.7%). The recurrence rate was the highest in peripheral giant cell granuloma (8, 12.3%), versus 6% in pyogenic granuloma and fibro-epithelial hyperplasia. These differences in the recurrence rate were statistically significant (*p* = 0.014). *Conclusions*: This study emphasizes the necessity of submitting tissue of peri-implantitis cases for histopathological analysis since the more locally aggressive lesions (peripheral giant cell granuloma and pyogenic granuloma), which comprise nearly half of the cases in this study, do not differ in clinical or radiographic characteristics from other peri-implant lesions.

## 1. Introduction

Peri-implantitis (PI) is a common condition involving soft tissue and bone surrounding dental implants. Its clinical characteristics include erythema, swelling, suppuration, recession, pocket formation, and loss of alveolar bone around the implant [1,2].

PI etiology is multifactorial. Predisposing factors include genetic factors, poor oral hygiene, surgical trauma during implant placement, inappropriate implant position, implant surface characteristics, inappropriate planning of the overlying prosthetic fixture, and overloading.

A key topic regarding the proposed etiology of peri-implantitis is the type of restoration used; numerous studies address the issue of cement-retained versus screw-retained restorations and marginal bone loss around implants. There is no consensus whether cement-retained implant-supported restorations show less marginal bone loss when compared with screw-retained restorations. Nevertheless, two recent studies demonstrated that cemented press-fit abutments showed a lower risk of peri-implantitis when compared with screwed abutments. This can be explained by a better seal of the implant-abutment connection, which reduces bacterial infiltration and micromovement [3,4,5,6,7].

Currently, there is no widely accepted protocol for the treatment of PI, and in many cases in which peri-implant tissue is removed during treatment, it is not submitted for histopathological analysis [2]. In our department, we take a biopsy from the peri-implant tissue when treating peri-implantitis cases surgically. Several previous histopathological studies of peri-implant tissue demonstrated the presence of only inflammation and hyperplasia [8,9,10,11,12,13,14,15]. In contrast, others showed that locally aggressive lesions were present in about half the cases analyzed (such as pyogenic granuloma, giant cell granuloma, and peripheral ossifying fibroma) [16,17]. These lesions did not differ in clinical or radiographic characteristics from other peri-implant lesions, which demonstrated only hyperplasia and chronic inflammation. Therefore, histopathological analysis of peri-implant tissue is essential for correct diagnosis. As yet, only a few studies report results of histopathological findings in PI [8,9,10,11,12,13,14,15,16,17,18,19,20,21], reporting together approximately 250 cases, possibly due to lack of clear treatment protocols for PI and, in particular, histopathological analysis not being an integral part of suggested protocols.

It is not clear from the existing literature what is the clinical implication of different histopathological diagnoses in lesions similar to PI. Questions that have not been widely addressed in the literature include the aggressiveness of these lesions, the association of any particular type with a higher risk for explantation or recurrence, the clinical differences in distribution, the association with systemic conditions, and the association between other clinical parameters in lesions mimicking PI with various histopathological classifications.

In rare cases, lesions mimicking PI, at least in early stages, represent either primary or metastatic malignancy [21].

Therefore, the objectives of the present study were to investigate the clinical and histopathological parameters of lesions mimicking PI and correlate them with the outcome and follow-up data, including recurrence and explantation rate. In addition, to investigate possible correlations with concomitant medications and systemic diseases.

## 2. Materials and Methods

A retrospective analysis of all consecutive biopsies taken from peri-implant tissues at the Oral and Maxillofacial Surgery Unit at the Tel-Aviv Medical Center between 2008–2019 was performed. The patients’ clinical data collected included demographical data, systemic medical conditions, medications used, histopathological diagnosis, length of follow-up, and data regarding recurrence and explantation.

The study was conducted according to the “Strengthening the Reporting of Observational Studies in Epidemiology” (STROBE) checklist [22] and received approval from the IRB committee (048-19-TLV).

Statistical analysis was performed using SPSS package, with chi-squared test or Fisher’s exact test for categorical variables.

## 3. Results

The study included 75 consecutive biopsies taken between 2008–2019 from 65 patients. The study population included 25 males and 40 females. The age range was 39–80 years (mean 66). In eight patients, two biopsies were taken, and in one patient, three biopsies. The statistical analysis included 65 biopsies and did not include additional biopsies from the same patients.

### 3.1. Follow-Up

Twenty-two (33.8%) patients were lost to follow-up. Of the 43 remaining cases, in 23 (53.5%) there was a follow-up period of less than 6 months, in 9 (21%) 6–12 months, in 6 (14%) up to 2 years, and 5 (11.6%) 2–5 years. The mean follow-up period was 8.5 months.

### 3.2. Pathological Diagnoses

**Figure 1:**
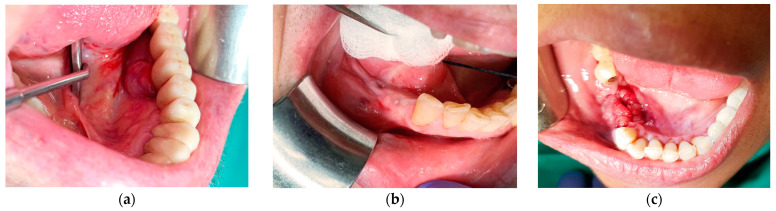
(**a**) A 74-year-old male with an exophytic mass on the lingual aspect of implant #36. The histopathological diagnosis was fibro-epithelial hyperplasia; (**b**) a 65-year-old female with a submucosal swelling surrounding a submerged implant #35. Two sinus tracts are evident on the buccal and lingual sides and one anteriorly to the site. The histopathological diagnosis was fibro-epithelial hyperplasia; (**c**) a 58-year-old female immediately after explantation of implants 45–46. The histopathological diagnosis was peripheral giant cell granuloma.

**Figure 2:**
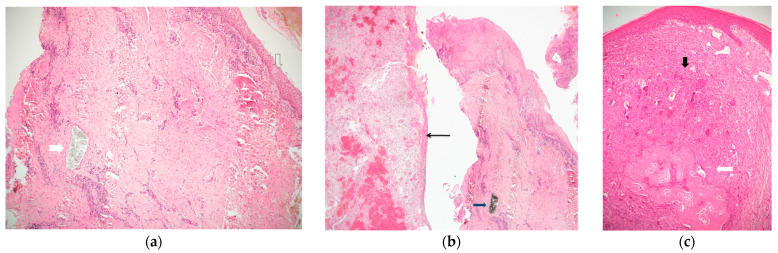
(**a**) A band of peri-implant tissue, lined by squamous epithelium (empty arrow), showing dense connective tissue and scattered chronic inflammation. The white arrow points to a collection of granular greyish foreign material, which does not elicit a foreign body type reaction. The diagnosis was inflammatory fibro-epithelial hyperplasia. (Hematoxylin and Eosin, original magnification ×40); (**b**) two bands of peri-implant tissue (from a single case). The right is lined by squamous epithelium, shows dense connective tissue, chronic inflammation, and a mass of granular black material (consistent with titanium shreds). The left side shows granulation tissue and is lined by thin non-keratinizing epithelium. The diagnosis was inflammatory fibro-epithelial hyperplasia and granulation tissue. (Hematoxylin and Eosin, original magnification ×40); (**c**) a mass lined by squamous epithelium, composed of clusters of multinucleated giant cells in a cellular and vascular matrix (black arrow), with an area of ossification (white arrow). The diagnosis was peripheral giant cell granuloma. (Hematoxylin and Eosin, original magnification ×40).

Results of histopathology of the cases included: fibro-epithelial hyperplasia (FEH) 20 (30.7%), pyogenic granuloma (PG) 16 (24.6%), peripheral giant cell granuloma (PGCG) 15 (23%), medication-related osteonecrosis of the jaws (MRONJ) 5 (7.7%), osteomyelitis 5 (7.7%), and one case (1.5%) each of ossifying fibroma, odontogenic cyst, oral squamous cell carcinoma, and foreign body reaction. Clinical and histological examples of lesions are exhibited in Figure 1 and Figure 2.

### 3.3. Location

The mandible was involved in 50 (77%) cases, while 13 (20%) were in the maxilla, and in 2 cases (3%), information on the location was missing. In both jaws there were significantly more lesions involving posterior rather than anterior areas (60% posterior mandible, 65% posterior maxilla).

### 3.4. Clinical Presentation

There were some inconsistencies in the recorded files regarding the features of the clinical presentation. Swelling and erythema were recorded in 43 (66%) cases, 14 (70%) in cases with FEH, 13 (81%) in PG, 10 (66.6%) in PGCG, 1 (20%) in MRONJ, and 2 (40%) in osteomyelitis.

Loss of bone (as a dichotomic parameter) was recorded in 31 (47.6%) cases, considering that in 19 (29.2%) cases, data on bone levels were missing in the files. In eight (40%) cases of FEH, bone loss was documented, seven (35%) did not show bone loss, and in five (25%) data was missing. In PG, five (31%) had bone loss, in four (25%) there was no bone loss, and in seven (44%) data was missing. In PGCG, eight (53%) had documented bone loss, two (13.3%) had no bone loss, and in five (33.3%) data was missing. In MRONJ, all five cases (100%) showed bone loss. In osteomyelitis three (60%) had documented bone loss, while two (40%) lacked bone loss.

A total of 43 (66%) cases presented with recorded swelling/erythema and bone loss, which are recognized signs of PI.

### 3.5. Recurrence

For the analysis of correlations between recurrence and pathological diagnosis, only the three most common diagnoses were included: FEH, PG, and PGCG. There were 18 cases of recurrent lesions in the study group (27.7%). The recurrence rate was the highest in PGCG (8, 12.3%), versus 6% in PG and FEH. These differences in the recurrence rate were statistically significant (*p* = 0.014).

### 3.6. Explantation

In total, 20 (30.7%) of all cases required explantation (implants were explanted during the biopsy). When explantation was compared by diagnostic groups, the lowest rate of three (18.7%) was found among cases with PG. In FEH, four (20%) cases had been explanted and in PGCG, four (26.6%). The highest rates were recorded in MRONJ, four (80%) and osteomyelitis, three (66%). However, these differences did not reach the threshold for statistical significance.

### 3.7. Use of Anti-Resorptive Medications

The use of various anti-resorptive medications was recorded in nine (13.8%) cases, of which five had been treated with oral bisphosphonates, one with IV-bisphosphonates, two with RANK-L inhibitors, and a combination of these in one case. MRONJ was documented in five of these cases, and correlation with these medications was not found in any of the other diagnostic groups. The etiology of all MRONJ cases presented here was peri-implantitis; osteonecrosis was not clinically overt before in these patients.

### 3.8. Correlation with Diabetes Mellitus

Diabetes mellitus was recorded in eight (12.3%) cases, evenly distributed between the various diagnostic groups.

### 3.9. Background of Non-Oral Malignancy

Non-oral malignancy was recorded in 12 (18.4%) cases of the study population, of which 9 (75%) had prostate cancer, and 1 (8.3%) each of breast cancer, non-Hodgkin lymphoma and polycythemia vera. Of these, five (41.6%) were diagnosed with MRONJ, four (33.3%) PG, and one (8.3%) each with PGCG, FEH, and foreign body reaction. These differences did not reach the threshold for statistical significance.

## 4. Discussion

The introduction of dental implants into the practice of dentistry has brought forward many advantages in patients’ function, esthetics, and quality of life. However, these advanced treatment modalities have also introduced a range of complications, the most common of which is PI. The characteristics of PI include erythema, swelling, and bleeding on probing. Suppuration may also be present. If changes are restricted to soft tissue, the term peri-mucositis is used, and if progressive loss of implant supporting bone is observed, the term PI is applied [8,23]. The reported frequency of PI varies between 12–43% of implant sites, and may reach 80%, when peri-mucositis as well as PI are included [24].

Without a universally accepted protocol, the treatment of PI varies. Although treatment of PI often includes surgical removal of peri-implant soft tissue, the tissues are rarely submitted for pathological analysis.

Previous studies have shown that when tissues from lesions suspected to be PI are submitted for pathological analysis, only 30–40% of cases showed inflammatory reactive fibro-epithelial hyperplasia. In the remaining cases, a variety of pathological entities were diagnosed, including PG, PGCG, peripheral ossifying fibroma (POF), and actinomyces-associated infection, all of which tend to be locally aggressive when present in the gingiva and bone around teeth. Primary malignancy of the oral mucosa as well as metastasis from non-oral malignancies have also been reported as histopathological diagnoses of peri-implant tissues. However, cases of peri-implant lesions belonging to this spectrum of benign and malignant possibilities tend to look clinically and radiographically indistinguishable from classical PI in many cases, and unless a biopsy is submitted, the correct diagnosis may be missed or delayed. In our study, one case was diagnosed as squamous cell carcinoma (1.5%).

The literature regarding PI includes only sparse information on the clinical behavior, recurrence, or failure rates of lesions such as FEH, PG, and PGCG around implants. The correlations of any of these lesions with possible predisposing conditions such as diabetes mellitus or use of anti-resorptive medications has also not been investigated before. The present analysis indicates that the rate of recurrent lesions depends significantly on the specific pathological diagnosis, with the highest recurrence associated with PGCG, with a lower risk in PG and FEH.

In previous studies of gingival pathology (unrelated to implants), the four most common diagnoses were FEH (55.9%), PG (26%), POF (10%), and PGCG (6.7%) [25,26].

In our previous study of peri-implant pathology [17], lesions presenting around implants showed a frequency of 10% PGCG and 18% PG. In the present study group, PGCG comprised 23% of lesions and PG 24.6%, together accounting for about 50% of biopsied cases. Compared with gingival lesions unrelated to implants, PG around implants has a similar frequency (26% vs. 24.6%, respectively). However, PGCG seems to be significantly more common around implants (23% vs. 6.7% in unrelated gingiva).

The present study was performed in collaboration with the oral and maxillofacial unit and the oral pathology service in a large tertiary referral center and combined clinical radiographic and pathological analysis and follow-up data. While comparing these data, in the context of a tertiary referral center, there may be some bias, as it is possible that the cases referred to oral and maxillofacial surgery unit may have been looking more atypical clinically than conventional PI or failed to respond to treatment. Another limitation is the lack of data regarding implant types, restorations and abutments used, and whether regeneration materials were used in conjunction with these implants.

## 5. Conclusions

All tissue removed from peri-implantitis cases should be submitted for microscopic analysis irrelevant of the status of disease, since the more aggressive lesions are indistinguishable clinically from common hyperplasia and inflammation.

## Data Availability

Data will be made available upon request to the corresponding author.
